# Optical‐Field‐Driven Electron Tunneling in Metal–Insulator–Metal Nanojunction

**DOI:** 10.1002/advs.202101572

**Published:** 2021-10-27

**Authors:** Shenghan Zhou, Xiangdong Guo, Ke Chen, Matthew Thomas Cole, Xiaowei Wang, Zhenjun Li, Jiayu Dai, Chi Li, Qing Dai

**Affiliations:** ^1^ CAS Key Laboratory of Nanophotonic Materials and Devices CAS Key Laboratory of Standardization and Measurement for Nanotechnology CAS Center for Excellence in Nanoscience National Center for Nanoscience and Technology Beijing 100190 P. R. China; ^2^ Center of Materials Science and Optoelectronics Engineering University of Chinese Academy of Sciences Beijing 100049 P. R. China; ^3^ Department of Electronic and Electrical Engineering University of Bath Bath BA2 7AY UK; ^4^ Department of Physics National University of Defense Technology Changsha 410073 P. R. China; ^5^ GBA Research Innovation Institute for Nanotechnology Guangzhou 510700 P. R. China

**Keywords:** high nonlinearity, MIM nanojunction, optical‐field‐driven tunneling, ultrafast electronics

## Abstract

Optical‐field driven electron tunneling in nanojunctions has made demonstrable progress toward the development of ultrafast charge transport devices at subfemtosecond time scales, and have evidenced great potential as a springboard technology for the next generation of on‐chip “lightwave electronics.” Here, the empirical findings on photocurrent the high nonlinearity in metal–insulator–metal (MIM) nanojunctions driven by ultrafast optical pulses in the strong optical‐field regime are reported. In the present MIM device, a 14th power‐law scaling is identified, never achieved before in any known solid‐state device. This work lays important technological foundations for the development of a new generation of ultracompact and ultrafast electronics devices that operate with suboptical‐cycle response times.

## Graphical abstract

The authors report on the high nonlinearity photocurrent in metal‐insulator‐metal nano‐junctions driven by ultrafast optical pulses in the strong optical‐field regime. A 14^th^ power‐law scaling is first achieved in the solid‐state device. This work lays important technological foundations for the development of a new generation of ultracompact and ultrafast electronics devices that operate with sub‐optical‐cycle response times.

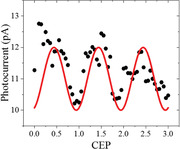

## 1 Introduction

The use of strong optical‐field in ultrashort pulsed lasers is today commonly employed in steering electrons at sub‐femtosecond time scales.^[^ ^1^, ^2^, ^3^, ^4^, ^5^, ^6^, ^7^, ^8^, ^9^, ^10^, ^11^, ^12^, ^13^, ^14^, ^15^ ^]^ More recently, interest has focused on utilizing such ultrafast electrons as carriers in solid‐state electronic devices due to their potential to overcome operation speed limitations plaguing present‐day electronics. Such approaches are widely heralded as a viable means of realizing a new generation of petahertz electronics.^[^ ^13^, ^14^, ^16^ ^]^ For this purpose, several fundamental types of optical‐field driven ultrafast electron devices have been proposed and demonstrated,^[^ ^17^, ^18^, ^19^ ^]^ among which nanojunction tunneling devices are perhaps some of the most promising given the case with which the strong optical‐field enhancement can be achieved in the nanojunction.

A key requirement for ultrafast devices is the need for highly nonlinearity, with a highly sensitive photocurrent modulation capable of sub‐femtosecond on–off transients. Previously reported ultrafast electron tunneling devices have suffered from relatively high vacuum barrier (commonly ≈ 5 eV),^[^ ^17^, ^18^, ^19^, ^20^, ^21^ ^]^ and when coupled to the need for access to the optical‐field driven regime only occurs at high field‐strength (>10 V nm^‐1^), has resulted in photocurrent nonlinearities reducing to very low values, typically ≈2.^[^ ^17^, ^20^, ^21^ ^]^ Although devices with this performance level have demonstrable potential in future petahertz electronics; specifically carrier‐envelope phase (CEP) detector,^[^ ^14^, ^22^ ^]^ this nonlinearity must be further increased in order to greatly enhance detection sensitivity.

Here, we report on the measured ultrafast laser‐driven photocurrent in a nanoscale MIM device. One of the advantages of the present MIM structure is that the tunneling barrier can be band‐optimized by selecting sandwiches created from a plethora of insulating and conducting materials.^[^ ^23^ ^]^ Compared to previously reported nano‐junction devices, the tunneling barrier of the present device is, as a result, greatly lowered to 1.73 eV, thereby enabling improved access to the optical‐driven tunneling regime at a much lower field‐strengthen of ≈5 V nm^‐1^.^[^ ^17^ ^]^ With the assistance of a DC bias voltage, the optical‐field‐driven tunneling regime has here been achieved with transport showing a high nonlinearity of up to 14 in the current–power (*I*–*P*) curve (*P* ^7^), a finding we further corroborate via polarization‐dependent experiments. This high nonlinearity constitutes an important leap in our understanding of transport in MIMs devices and contributes toward the realization of ultracompact and ultrafast PHz solid states electronics devices, such as CEP detectors.

## 2 Results and Discussion

**Figure 1:**
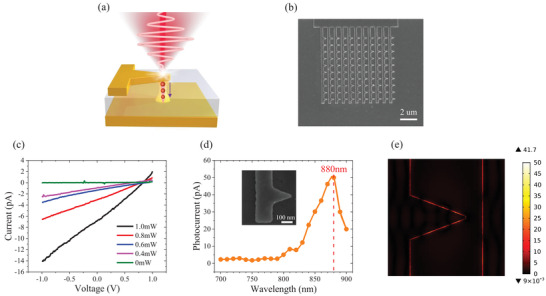
Typical fabricated antenna coupled metal‐insulator‐metal (MIM) nanojunction. a) Schematic depiction of the MIM device with electron emission being stimulated by a femtosecond laser pulse. b) A scanning electron microscope (SEM) image of a representative fabricated MIM device. (Scale bar: 2 µm). c) Typical measured *I*–*V* characteristics of a MIM device without laser illumination (0 mW) and as a function of different laser power. d) Photocurrent spectra of the MIM device with different laser wavelengths. The measured photocurrent spectrum has a peak near 880 nm. Bias voltage: ‐2 V, laser power: 1 mW. Inset: SEM image of the measured device. (Scale bar: 100 nm). The width of the stripe is ≈300 ± 30 nm, the base of the nanotriangle is ≈300 ± 20 nm, and the height is ≈400 ± 30 nm. e) Simulated electromagnetic field distribution in Ti/Au‐Al_2_O_3_ interface of the MIM device. The maximum of field enhancement is 41.7 at *λ* = 880 nm.

Similar to structures formed elsewhere,^[^ ^17^, ^23^ ^]^ the present MIM structure, as shown in **Figure** **1**a, consists of a top electrode formed from a 3/60 nm Ti /Au layer, followed by an insulator formed from an 8 nm‐thick ALD‐grown nano‐Al_2_O_3_ film, and finally a 60 nm thick Au layer which forms the bottom electrode. Using electron‐beam lithography, the top electrode is subsequently shaped into nanotriangle structures of 300 × 400 nm, which serve to focus the free‐space optical field to a nanoscale hotspot at the nanotriangle tip. Figure 1a depicts the photoemission process of a single unit of the asymmetric MIM structure excited by a femtosecond laser pulse. A typical fabricated device is shown in Figure 1b. The focused optical field is then coupled to the MIM structure to achieve a high field enhancement factor within the nanogap. A linear polarized, ultrafast laser with an FWHM pulse width of 140 fs, 80 MHz repetition rate, a tunable wavelength (680–1050 nm), and a spot size of ≈2.5 µm (FWHM), was illuminated vertically onto the sample surface. Photoexcited electrons were then attracted by the combined effects of a DC electric field and the incident optical field, with liberated electrons emitted from one electrode to the counter electrode. Although the photocurrent is nominally bidirectional, in the present work we focus on exploring those photocurrents from the top nanotriangular structures to the bottom flat electrode in order to investigate the field enhancement impacts of the top nanotriangle electrode (Figure S1a, Supporting Information). To obtain a measurable photocurrent, the top electrode consists of a series of parallel Au stripes (the laser irradiation area ≈3 µm) with asymmetric nanotriangle structures and the total measured current is the sum of all photoexcited nanotriangle photocurrent contributions.

As shown in Figure 1c, we measure both the tunnel current *I*_dc_ without laser illumination and the photoemission current *I*_emission_ during laser excitation as a function of different optical power (*P*). The DC current without laser illumination shows no measurable signal (*I* < 0.1 pA, commensurate with the noise floor of the precision ammeter used). However, a remarkable current–voltage (*I*–*V*) characteristic evolves with increasing laser illumination. The device rectifies, an effect which we attribute to the asymmetric in the junction's material composition (Ti/Au‐Al_2_O_3_‐Au). The zero current point is at around 0.8V, suggesting a Schottky barrier height difference of 0.8 eV. This is consistent with values reported elsewhere as well as our measured work function difference between the Au (5.1 eV) and Ti (4.33 eV) in the present structure.^[^ ^24^, ^25^ ^]^ The wavelength‐dependent photocurrent spectrum (Figure 1d) suggests a plasmon resonant peak of 880 nm for the given device geometry, as shown in the inset of Figure 1d. Figure 1e shows the simulated electromagnetic field distribution in the Ti/Au‐Al_2_O_3_ interface of the MIM device at *λ* = 880 nm. The field enhancement at 880 nm (*β* = 41.7) is notably enhanced compared to that at 730 nm (Figure S1b, Supporting Information, *β* = 27.6), corresponding to the photocurrent spectrum shown in Figure 1d.

**Figure 2:**
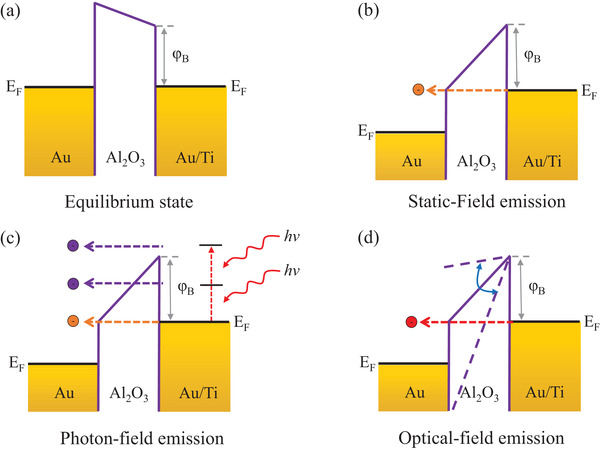
MIM nanojunction operation principle. a) Energy band diagram for the equilibrium state (no bias, no illumination). Where *φ*_B_ is the barrier height (≈1.73 eV), *E*_f_ is the Fermi level. b) Energy band diagram during static‐field emission. Depending on the bias voltage, the static‐field‐driven tunneling may transit from direct tunneling (rectangular barrier) to F–N tunneling (triangular barrier). c) Energy band diagram for photon‐field emission, including multiphoton photoemission and photo‐assisted tunneling. *hv* is the photon energy. d) Energy band diagram for optical‐field emission.

The MIMs operation principle is illustrated in **Figure** **2**. The equilibrium state (no bias, no illumination) is shown in Figure 2a. The barrier height is approximately equal to the difference in the work function for Ti (*W*_Ti_ = 4.33 eV) and the electron affinity of the crystalline Al_2_O_3_ (*χ*_Al2O3_ = 2.6 eV), that is, *φ*_B_ = 1.73 eV. When biased, but without laser illumination, the device operates in the conventional static‐field‐driven electron tunneling regime.^[^ ^26^, ^27^ ^]^ Dependent on the bias voltage, this static‐field‐driven electron tunneling may transit from direct tunneling (rectangular barrier) to F–N tunneling (triangular barrier, Figure 2b). Under ultrafast laser illumination at relative weak power (<0.3 mW), photon‐assisted tunneling dominates^[^ ^28^ ^]^—electrons around the Fermi level absorb one or more photons exciting them into a higher energy level and then tunnel into the conduction band of Al_2_O_3_, assisted by the static‐field. When driven at medium optical powers (<0.6 mW), the device operation adopts multiphoton photoemission^[^ ^21^, ^29^, ^30^, ^31^ ^]^—electron around the Fermi level (*E*_f_) absorb additional photons and in doing so obtain sufficient energy to overcome the surface barrier entirely (Figure 2c). In the present device structure, as the Schottky barrier height is ≈1.73 eV, one‐photon‐assisted tunneling and two‐photon photoemission likely dominate under the optical pulsed excitation. When illuminated with a much more intense laser (>0.6 mW), the strength of the optical‐field in the nanojunction is likely sufficiently high (≈5 V nm^‐1^) to perturb the Schottky barrier such that the electrons around *E*_f_ may tunnel through the periodically narrowed barrier as a result of the oscillating optical‐field (Figure 2d).^[^ ^13^, ^14^, ^32^, ^33^ ^]^

**Figure 3:**
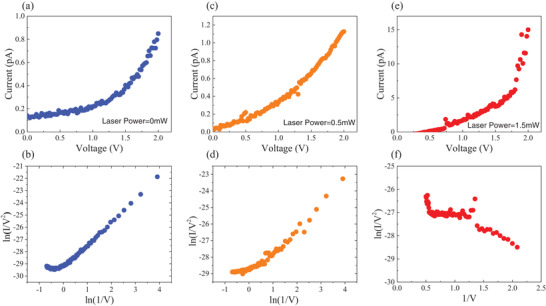
Transport characteristics as a function of laser power. Measured *I*–*V* curve a) without laser illumination (0 mW), and with optical power of c) 0.5 mW, and e) 1.5 mW. (b,d,f) are the corresponding F–N plots, respectively.

An example of a typical *I*–*V* curve measured for devices illuminated with different laser powers is shown in **Figure** **3**. Figure 3a shows the tunneling behavior without laser excitation. Below 1 V, the *I*–*V* curve shows widely reported linear behavior associated with conventional direct tunneling.^[^ ^34^, ^35^ ^]^ Conversely, at the higher bias (>1 V), the tunneling behaves increasingly nonlinear, adopting more F–N‐like tunneling as one would expect for an electron‐dense emitting surface. This is further evidenced by the F–N fitting of the *I*–*V* curve, as shown in Figure 3b. When exposed to optical pulsing at moderate intensity (≈7 × 10^8^ W cm^‐2^), the tunneling behavior changes dramatically. The *I*–*V* curve becomes increasingly linear (Figure 3c) and the developed high F–N nonlinearity at high bias (>1V) disappears (Figure 3d). This suggests direct tunneling is not dominant, but the multiphoton photoemission^[^ ^29^, ^30^ ^]^ becomes increasingly dominant, with this transition shown in Figure 3d, we will discuss this photo‐driven process after. In the photon‐driven regime, the *I*–*V* curve should become increasingly linear as the barrier is undisturbed.^[^ ^36^ ^]^ However, at optical power densities >1.4 × 10^9^ W cm_‐_^2^, we observe a clear deviation from this photon‐driven regime, with the measured *I*–*V* profiles adopting an unexpected (Figure 3e) and more F–N‐like tunneling regime (*V* > 1 V) (Figure 3f). F–N tunneling is induced by a strong electric field and should not be solely induced by the bias voltage according to our findings in Figure 3a. We attribute this anomalous behavior to a new field‐driven tunneling mode created by the superposition of static‐ and optical fields.

**Figure 4:**
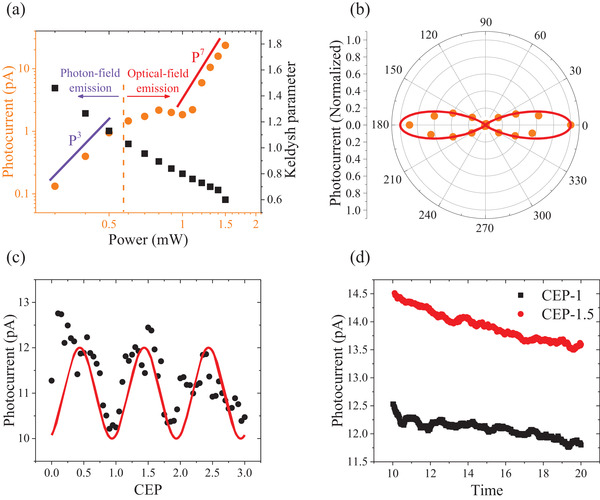
MIM nanojunction photon response. a) Laser‐induced tunneling current (orange points) and calculated Keldysh parameter (black points) as a function of increasing laser power at the resonant wavelength (880 nm). b) Photocurrent polarization‐dependent at *λ* = 880 nm (orange points), exhibiting a cos^14^(*θ*) angular dependence (red line). Angle 0° of polarization is parallel to the height of the nanotriangle. c) Laser‐induced tunneling current (black points) and fitting cos(*x*) curve (red lines) as a function of different laser CEP. d) Laser‐induced tunneling current when the laser CEP is 1 and 1.5, respectively.

To confirm the optical‐field‐driven mechanism observed, the power‐dependent photocurrent, at a fixed bias voltage (+2 V), was measured. Here we observed two distinct photoemission behaviors at the resonant and nonresonant wavelength. At a nonresonant wavelength of 730 nm, the photoemission current—light field strengthen (*I*–*P*) curve shows a known power‐law dependence (*I* ∼ *P* ^2^), as reported elsewhere, indicating multiphoton photoemission^[^ ^29^, ^30^ ^]^ (Figure S2, Supporting Information). However, at the resonant wavelength of 880 nm, the *I*–*P* curve adopts a dramatically different form from the expected power‐law scaling. As shown in **Figure** **4**a, the *I*–P (orange points) adopts three distinct regimes. At low power (<0.6 mW), the profile is nearly a third‐order power‐law scaling (purple line in Figure 4a, *I* ∼ *P* ^3^). As one electron is required to absorb just two photons (photon energy, 1.41 eV) to overcome the Schottky barrier (1.73 eV), this will result in a two‐order power‐law scaling, the photo‐driven tunneling is not a pure multiphoton photoemission process. We attribute the third power‐law scaling to a possible hybrid mechanism, which involving two‐photon photoemission, one‐photo‐assisted tunneling and direct tunneling from resonantly excited states (Figure 2c). Moreover, when the laser is irradiated, the electrons in the gold valence band may also be excited, which may increase the power‐law scaling. At medium power (<1 mW), the curve deviated from this three‐photon scaling to a lower power order scaling (≈1), with a slight decrease (≈10%) in the measured photocurrent. The space‐charge effect may account for this reduction.^[^ ^37^, ^38^ ^]^ However, as the photocurrent is <12 pA, which corresponds to less than one electron per pulse, and as the photocurrent increases again at elevated powers (1.2 mW), it is likely that the space‐charge effect can be eliminated given the low charges density involved. We will discuss the mechanism of this flat region in detail later. We noted that, following the decrease in photocurrent, at higher power (>1.2 mW), the profile behaves much more nonlinearly and tends to an *I* ∼ *P* ^7^ order (red line, Figure 4a) that is here observed for the first time. This high nonlinearity is corroborated in Figure 4b by polarization dependence photocurrent measurements (orange points) where the date can be fitted to a cos^14^(*θ*) (red line). Here, the Keldysh parameter (black points in Figure 4a) was used to further estimate the magnitude of the optical‐field required to support the quasistatic electron tunneling, and is givenby^[^ ^39^ ^]^

(1) $$\gamma =\omega \sqrt{2m^{*}\phi }/e\beta F_{0}$$

where *ω* is optical frequency, *φ* is the height of Schottky barrier, m* is the effective mass of the electron (*m*_0_ is the free electron mass) and *e* is its charge, *F*_0_ is the incident optical‐field strength, and *β* is the optical‐field enhancement factor of the nanogap of the MIM structure. Recently, it has been observed that the transition to tunneling behavior occurs when *γ* = ≈2.^[^ ^17^, ^20^ ^]^ In this work, m* = 0.25 *m*_0_,^[^ ^25^ ^]^ for a 1.4 × 10^9^ W cm^‐2^, 880 nm incident laser, the calculated *γ* is ≈0.8, further supporting the optical‐field‐driven electron tunneling regime observed.

The deviation of the *I*–*P* curve from power‐law scaling has been widely investigated both experimentally and theoretically.^[^ ^17^, ^20^, ^40^, ^41^ ^]^ Near the transition from power‐law scaling (the intermediate transitional region between the slope = 3 and the slope = 7 regimes in Figure 4a), the strength of the optical field is such that it readily disturbs the Schottky barrier. This induces the multiphoton channel to close,^[^ ^41^ ^]^ thereby reducing the nonlinearity, leading to the power‐law scaling with a lower slope of 1.^[^ ^33^ ^]^ This is consistent with the experimental results in the present work. Moreover, the fluctuation phenomenon of the *I*–*P* curve has been theoretically associated with the interference of electron wavepackets that coherently emit from two adjacent optical‐cycle of the laser pulse.^[^ ^37^, ^38^ ^]^ It should be noted that this only occurs in the optical‐field‐driven regime. However, such an *I*–*P* fluctuation phenomenon has seldomly been experimentally observed, primarily as a result of electron wavepacket decoherence, driven by environmental factors linked to necessarily long‐distance propagation. The present MIMs device structure benefits from nanoscale propagation distances of <10 nm, allowing for stable access to the coherent nature of the optical‐field‐driven tunneling electrons, and thus, the coherent phenomenon can be observed.

In order to verify the optical‐field driven tunneling process, we also measured the CEP effect of the photocurrent with 7 fs laser pulse. As shown in Figure 4c, the photocurrent (black points) exhibits a cos(*x*) periodic CEP modulation effect (red lines) with different laser CEP. A clearly CEP‐dependent tunneling current with a modulation depth of up to 20% is observed. Figure 4d shows the measurement photocurrent extracted from Figure 4c when the laser CEP is 1 and 1.5 respectively. The photocurrent modulated by laser CEP is higher than the photocurrent decay with the same laser CEP, which showing the CEP modulation effect and indicated the optical‐field driven tunneling process in the present MIM device.

The present MIM nanojunctions can be optimized by reducing the Schottky barrier further by incorporating additional low work function materials. Beyond conventional electrode materials, some 2D electrode materials^[^ ^42^ ^]^ and 3D topological semimetals^[^ ^43^ ^]^ are emerging candidates for light‐based device applications. There are also further opportunities in using other dielectric materials such as 2D h‐BN in order to further enhance the local fields. With the continued reduction in the gap dimensions, as well as an increased wavelength, such systems will provide a unique platform upon which to explore the transition of nanogap electron propagation from the quiver to the subcycle regime. The present device also provides a promising means of constructing two barrier structures, and thus a way to realize some of the first optical‐field‐driven resonant tunneling structures.

## 3 Conclusion

Here we have explored the detailed transport behavior in ultrafast laser‐driven tunneling currents through a vertically constricted MIM tunneling junction with dimensions of just a few nanometers. The measured *I*–*P* properties firmly indicate a high nonlinearity up to 14 which suggests novel intercycle electron packet interference. These results demonstrate that MIM structures afford a unique platform upon which to establish, measure, and manipulate coherent electron tunneling driven by optical fields. These findings represent encouraging progress toward a new class of electronic nanodevices that operate via finely tunable electron dynamics. Such MIMs systems and the transport they support contribute promising research and practical directions in the ongoing efforts to shrink electronics ever further. These findings highlight that optical‐field‐driven inelastic tunneling may be a promising approach for realizing attosecond light sources with these experimental observations motivating future theoretical efforts to describe mesoscopic electron systems at the intersection between attosecond optics and quantum plasmonics.

## Conflict of Interest

The authors declare no conflict of interest.

## Supporting information

Supporting Information

## Data Availability

Research data are not shared.
