# Supplementary material for: Optical‐Field‐Driven Electron Tunneling in Metal–Insulator–Metal Nanojunction
Source: Adv Sci (Weinh). 2021 Oct 27;8(24):2101572. doi: 10.1002/advs.202101572 (PMC8693043; doi:10.1002/advs.202101572)
Supplement: Supplementary file 1 — Supporting Information [file ADVS-8-2101572-s001.pdf]

WILEY-VCH

© 2021 Wiley-VCH GmbH

ADVANCED SCIENCE
Open Access

Supporting Information

for *Adv. Sci.,* DOI: 10.1002/advs.202101572

Optical-Field-Driven Electron Tunneling in Metal-Insulator-Metal Nano-junction

Shenghan Zhou, Xiangdong Guo, Ke Chen, Matthew Thomas Cole, Xiaowei Wang, Zhenjun Li, Jiayu Dai, Chi Li, and Qing Dai**

Supporting Information

R1: MIM nano-junction fabrication

Electron beam lithography (EBL) was used to fabricate the MIM nano-junctions on 525 (±25) μm thick silicon (phosphorus doping, 1-10 ohm-cm) substrate coated with a 285 (±20) nm SiO_2 layer (thermal oxide). This was achieved using a positive electron beam resist (polymethylmethacrylate (PMMA) with a molecular weight of 950 K) that was spin-coated at 4000 rpm for 60 s which was soft-baked for 120 s at 180 °C. The bottom electrode was similarly patterned by EBL at 100 kV and an exposure dose of 900 $\mu C/cm^2$. Resist was developed by rinsing the exposed samples in a mixture of 3:1 isopropanol alcohol (IPA) and methyl isobutyl ketone (MIBK) for 60 s, and then fixing in IPA for 30 s. A 60 nm thick Au layer was deposited by electron-beam evaporation (OHMIKER-50B). The PMMA resist layer was lift-off removed in acetone (≥99.5%, Sinopharm Chemical Reagent Co., Ltd.) for 20 min in a heated water bath at 80 °C. This electrode was then overgrown with an 8 nm-thick Al_2O_3 layer by atomic layer deposition (ALD, SENTECH Instruments GmbH). Finally, the above EBL process was repeated to fabricate the array of nanotriangle antennas above the bottom electrode. The top electrodes consisted of a series of 11 parallel Au stripes (3 nm Ti/60 nm Au) with asymmetric nanotriangle structures, which are oriented in an orthogonal direction of the bottom electrodes. The base of the nanotriangle is h ~ (300 ± 20) nm, with height is l ~ (400 ± 30) nm.

R2: Characterization and electronic measurements

The morphologies of the MIM nano-junctions were characterized by SEM (FEI NOVA nano-430). The I-V characteristics of the MIM nano-junctions were measured with Keithley 2636B source measure unit. Incident light from a femtosecond laser source (Chameleon Ultra Laser System, Coherent) was focus on the sample using 50× Olympus objective (0.55 NA, 8.2 WD) resulting with an FWHM spot size of ∼2.5 μm. Optical images were obtained under the same objective and recorded using a CCD camera (MER-310-12UC, Daheng Optics) to confirm the laser position on the antennas. All measurements were performed at atmospheric pressure at room temperature.

R3: Simulations

Finite element simulations were conducted in the COMSOL software to investigate the electromagnetic field distributions and field enhancement with the MIM nano-junctions.

R4:

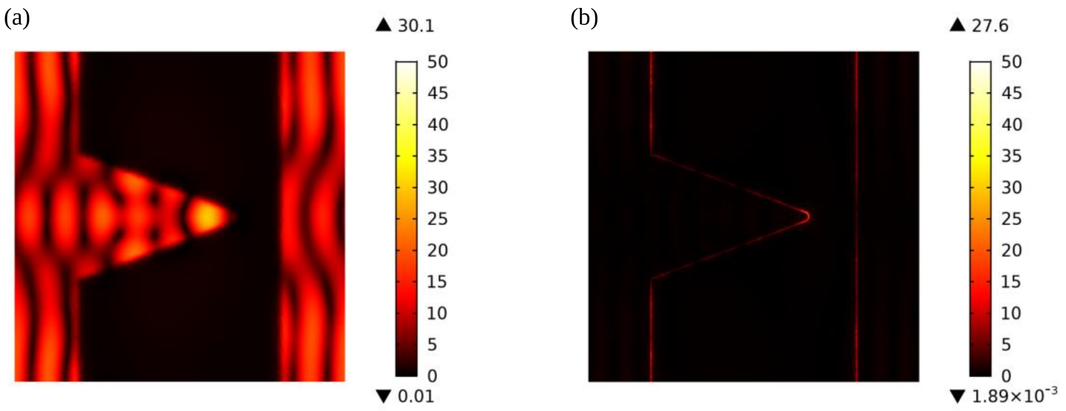

Figure S1. (a) Electromagnetic field distribution at the Al_2O_3-Au interface. The maximum of field enhancement (β=30.1) is lower than the Ti/Au-Al_2O_3 interface at λ=880 nm. (b) Electromagnetic field distribution at the Ti/Au-Al_2O_3 interface of the MIM device, with a maximum field enhancement of 27.6 at λ=730 nm.

R5:

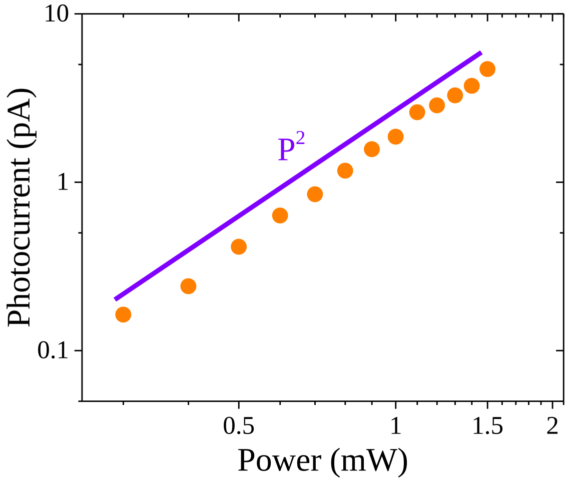

Figure S2. Laser-induced tunneling current as a function of increasing laser power at the non-resonant wavelength (730 nm). The curve is a two-order power-law scaling (purple line, $I \sim P^2$), corresponding multiphoton photoemission.

R6:

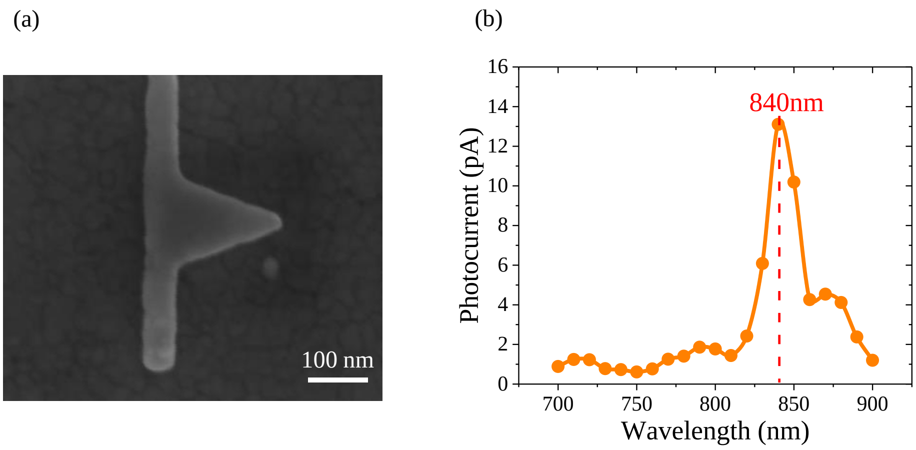

Figure S3. (a) SEM image of a single fabricated MIM device formed with 100 nm width stripes. (b) Photocurrent spectra of this MIM device as a function of different laser wavelengths, demonstrating a resonant peak near 840 nm.

R7:

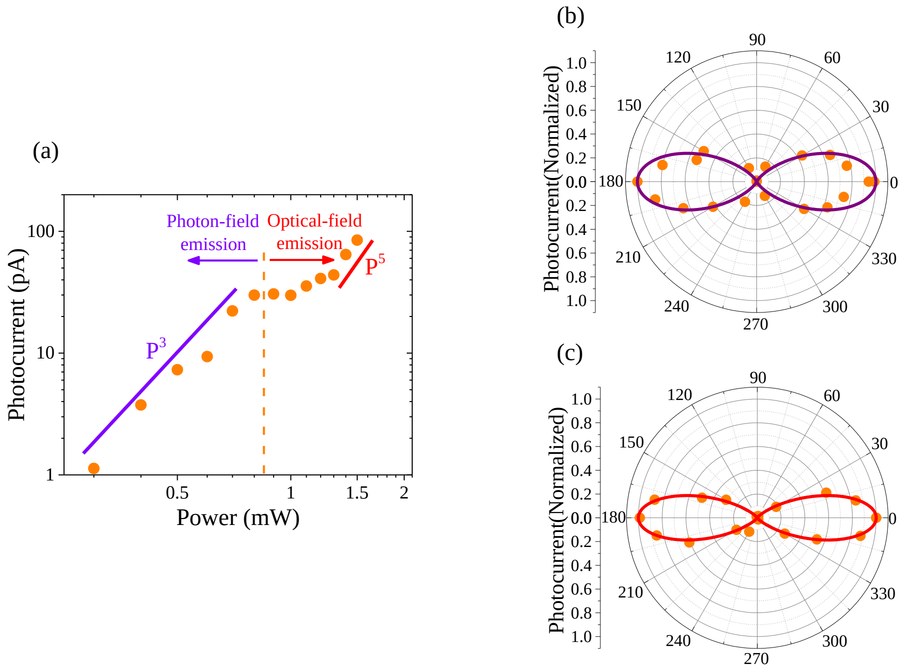

Figure S4. (a) Laser-induced tunneling current as a function of increasing laser power at 840 nm. The I-P curve behaves similar characteristics with three different regimes. (b) Photocurrent polarization-dependent at λ=840 nm at 0.5 mW (orange points), exhibiting a $\cos^6(\theta)$ angular dependence (purple line). Angle 0° of polarization is parallel to the height of the nanotriangle. (c) Photocurrent polarization-dependent at λ=840 nm at 1.4 mW (orange points), exhibiting a $\cos^{10}(\theta)$ angular dependence (red line).
